# Predicting the spread of invasive *Imperata cylindrica* under climate change: A global risk assessment and future distribution scenarios

**DOI:** 10.1371/journal.pone.0321027

**Published:** 2025-05-09

**Authors:** Seyedeh Parvin Hejazi Rad, Tayna Sousa Duque, S. Luke Flory, Vanessa Gonçalves do Nascimento, Debora Sampaio Mendes, Josiane Costa Maciel, José Barbosa dos Santos, Ricardo Siqueira da Silva, Farzin Shabani

**Affiliations:** 1 Departamento de Agronomia, Universidade Federal dos Vales do Jequitinhonha e Mucuri, Diamantina, Minas Gerais, Brazil; 2 Agronomy Department and Invasion Science Institute, University of Florida, Gainesville, Florida, United States of America; 3 Departamento de Agronomia, Universidade Federal dos Vales do Jequitinhonha e Mucuri, Diamantina, Minas Gerais, Brazil and Invasion Science Institute, University of Florida, Gainesville, Florida, United States of America; 4 Departamento de Agronomia, Universidade Federal dos Vales do Jequitinhonha e Mucuri, Diamantina, Minas Gerais, Brazil and Department of Ecological Modelling, Helmholtz Centre for Environmental Research—UFZ Leipzig, Leipzig, Germany; 5 College of Arts and Sciences, Qatar University, Doha, Qatar; Universidade de Coimbra, PORTUGAL

## Abstract

Invasive plant species, such as *Imperata cylindrica* (cogongrass), threaten native ecosystems, natural resources, and lands worldwide. With climate change, the risk of invasions may increase as more favorable conditions enable non-native species to spread into new areas. This study employs the CLIMEX model to predict the potential distribution of *I. cylindrica* under current and future climate scenarios, under the SRES A2 scenario. A comprehensive dataset comprising 6,414 occurrence records was used to simulate the species’ ecological niche based on key climatic parameters, including temperature and soil moisture. Our results indicate that more than 16% of the global land surface is currently highly suitable for *I. cylindrica* (Ecoclimatic Index ≥ 30), with significant risk areas identified in Central America, Africa, and Australia. Future projections under the A2 scenario suggest an expansion of suitable habitats by 2050, 2080, and 2100, particularly in regions such as southern Argentina and parts of North America, while areas in Africa may experience a decrease in suitability due to rising temperatures. Sensitivity analysis revealed that temperature-related parameters (DV0, DV1, DV2, and DV3) are the most influential in determining the species’ distribution, highlighting the critical role of climate in driving the invasive potential of *I. cylindrica*. These findings provide valuable insights into the future risks associated with *I. cylindrica* invasions.

## Introduction

Environmental changes are driven by at least three interacting factors: climate change, anthropogenic activities, and biological invasions [1,2]. Climate change has a profound impact on the functioning of ecosystems, societies, and economies [3], altering the growth patterns of various organisms [4] and significantly contributing to biological invasions [5]. Elevated CO₂ concentrations in the environment increase the competitiveness of invasive plants compared to native species [6,7]. Moreover, increases in temperature, greenhouse gas emissions, and alterations to hydrological cycles [8] can induce phenotypic plasticity, which has been reported as a facilitator of invasions [9]. In this context, the long-term management of invasive plants must include spatially explicit projections of how global changes influence the risk of invasion.

Ecological niche modeling is a promising approach for predicting risk areas, which identifies regions characterized by climatic conditions suitable for a species’ distribution [10]. Accurately delineating a species’ geographical distribution is rarely achieved and depends on a complex interaction of various factors [11], but the climate is highly relevant at regional and continental scales [12]. Among the tools available for ecological niche projection, Species Distribution Models (SDMs) have proven effective in predicting the potential distribution of species using occurrence and climatic data [13]. Despite their utility and versatility, few invasive species have been subjected to ecological niche modeling, particularly in tropical regions such as Africa and Brazil, which host some of the world’s greatest biodiversity [14].

This study focuses on the species *Imperata cylindrica* (L.) Beauv., invasive in several countries and ranked as the seventh most problematic perennial plant globally, negatively affecting 35 crops in 73 tropical regions and causing significant economic and environmental damage [15]. The rapid invasiveness of *I. cylindrica* is driven by its rhizomes, which serve as the primary mechanism of regeneration and dissemination [16]. The invasiveness of *I. cylindrica* presents significant challenges for its control, requiring approaches that integrate preventive, cultural, mechanical, and biological methods [17].

*Imperata cylindrica* has previously been modelled using bioclimatic envelopes based on the Maxent [18] and Mahalanobis Distance (MD) models, under the A1B SRES scenario, for the United States of America [19] and India [20]. However, the results indicate the need for new models because the current ones are limited and may underestimate the species’ invasive potential [19]. These models do not integrate biological processes into their predictions, while the mechanistic modeling employed in the CLIMEX software correlates species occurrence with climate-related growth and stress parameters [21–24]. Additionally, using different scenarios allows for determining how different trajectories may influence the biological invasion of species.

Given the severity of the invasion of *I. cylindrica* and considering the knowledge gap regarding its spatial distribution and adaptation in different regions of the world under various climate change scenarios, this study aims to predict climatically suitable areas for the species using the CLIMEX software under the SRES A2 scenario. Furthermore, our study seeks to understand the species’ distribution dynamics in response to predicted climate changes and to identify the key climatic parameters in determining its potential distribution.

## Materials and methods

This study utilized CLIMEX (version 4.0, Hearne Software, Melbourne, Australia) to generate predictive distribution maps for *I. cylindrica* under current and future climate scenarios. CLIMEX is a mechanistic modeling software designed to assess habitat suitability for a specific species by integrating climatic and physiological data with known distribution patterns [25]. CLIMEX was chosen because the species’ biology influences the process of biological invasion, and various studies have already demonstrated the software’s efficiency in predicting potentially invaded areas [26,27].

**Fig 1:**
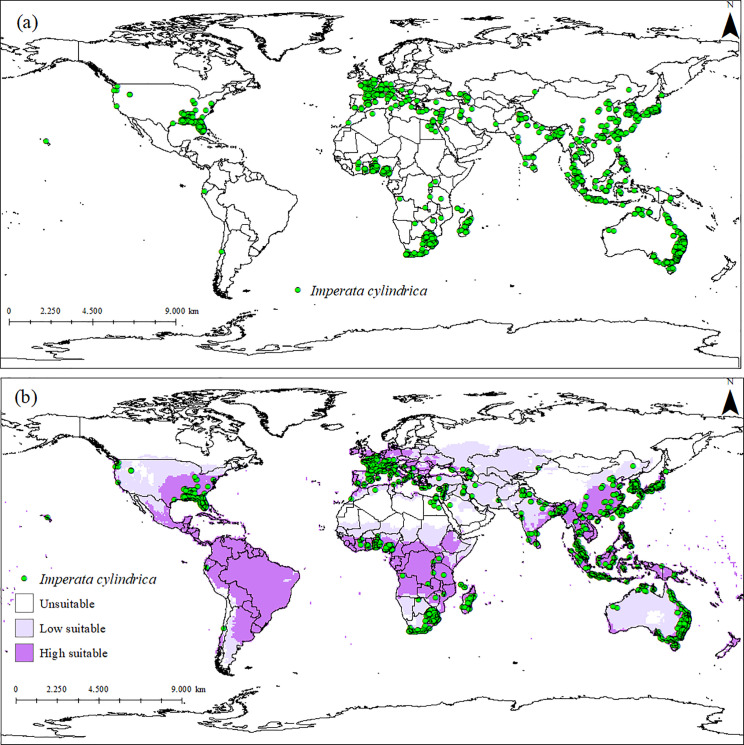
(a) Global distribution of *Imperata cylindrica* and (b) Ecoclimatic Index (EI) of *I.* ***cylindrica*, modeled using CLIMEX.** Unsuitable areas in white (EI = 0), low-suitability areas in purple (0 <EI <30), and high-suitability areas in purple (30 ≤ EI ≤ 100).

For modeling *I. cylindrica*, the first step involved obtaining distribution data from the Global Biodiversity Information Facility [28] and the Atlas of Living Australia [29], supplemented with additional occurrence records extracted from the scientific literature. The total number of records amounted to 6414 points, primarily distributed in tropical regions. Alongside the distribution data collection, we obtained information on ideal and limiting climatic conditions for the growth of *I. cylindrica* (Fig 1).

The CLIMEX software integrates these data into growth and stress indices, which determine the Ecoclimatic Index (EI) (Equation 1) [25,30]. The EI ranges from 0 to 100, with areas scoring above 30 considered highly suitable for the species’ occurrence [31]. This index identifies regions with favorable climatic conditions and highlights factors that may limit the potential distribution of *I. cylindrica*.


$$EI={GI}_{A\;}\times SI\;\times SX$$ (1)

Where: EI: ecoclimatic index; GIA: annual growth index; SI: annual stress index; SX: interaction between stress indices.

For *I. cylindrica*, the minimum temperature threshold for survival (DV0) was set at 5°C [32] as germination only occurs above this temperature. The optimal temperatures (DV1 and DV2) were set based on studies by Ekeleme et al. (2004) [33], Daneshgar et al. (2008) [34], and Daneshgar et al. (2009) [35], which showed vigorous growth of *I. cylindrica* in the range of 18°C to 24°C, respectively. The maximum temperature threshold (DV3) was established at 45°C [32,36] (Table 1).

**Table 1:** CLIMEX parameter values used for *Imperata cylindrica* modeling.

Index	Parameter	Values
Temperature	DV0 = lower threshold DV1= lower optimum temperature DV2 = upper optimum temperature DV3 = upper threshold	5°C 18°C 24°C 45 °C
Moisture	SM0 = lower soil moisture threshold SM1 = lower optimum soil moisture SM2 = upper optimum soil moisture SM3 = upper soil moisture threshold	0.07* 0.25* 1.5* 2.5*
Cold stress	TTCS = temperature threshold THCS = stress accumulation rate	5°C -0.0001 week ^-1^
Heat stress	TTHS = temperature threshold THHS = stress accumulation rate	45°C 0.1 week ^-1^
Dry stress	SMDS = soil moisture threshold HDS = stress accumulation rate	0.07* -0.0001 week ^-1^
Wet stress	SMWS = soil moisture threshold HWS = stress accumulation rate	2.5* 0.0025 week^-1^
Degree days	PDD = degree days per generation	1602 days

*Values without units are dimensionless indices of 100 mm single bucket soil moisture model (0 = oven – dry, 1 = field capacity).

The temperature threshold for cold stress (TTCS) was set at 5°C, aligning with DV0, and the rate of cold stress accumulation (THCS) was defined as -0.0001 week⁻¹, indicating the dormancy of *I. cylindrica* seeds when exposed to low temperatures. The temperature threshold for heat stress (TTHS) was set at 45°C, according to DV3 [32]. The heat stress accumulation (THHS) rate was established at 0.01 week⁻¹. The threshold for hot-dry stress (TTHD) was set at 45°C, the moisture threshold for hot-dry stress (MTHD) at 0.01 SMC, and the rate of hot-dry stress accumulation (PHD) at 0.01 week⁻¹ (Table 1). The species’ physiological mechanisms demonstrate a high sensitivity to thermal stress, and the thermal tolerance of photosynthetic tissues indicates the plant’s capacity to adapt to the challenges of a progressively warming climate [37].

The lower soil moisture threshold (SM0) was set at 0.07, with optimal soil moisture levels established at 0.2 (SM1) and 1.5 (SM2), while the upper soil moisture threshold (SM3) was adjusted to 2.5. These soil moisture thresholds were adapted to reflect conditions typical of regions such as Iran and Pakistan, where *I. cylindrica* thrives. The broad range of soil moisture conditions reflects the species’ adaptability to arid environments and its phenotypic plasticity, including increased aerenchyma formation in flooded areas [38].

The drought stress threshold (SMDS) was set at 0.05, with the rate of water stress accumulation (HDS) adjusted to -0.005 week⁻¹, considering the arid regions where *I. cylindrica* is found in the Sonoran Desert and parts of Africa. The moisture stress parameter (SMWS) was set at 0.05, and the accumulation rate (HWS) at 0.0025 week⁻¹. Drought and moisture stresses were incorporated into the model because, despite the species’ phenotypic plasticity, specific genotypes are absent in extremely dry or waterlogged areas [38,39]. The degree days required for developing *I. cylindrica* were set at 1602 (Table 1).

Modeling was conducted using the Climond 10-minute grid climatic data. These files cover the period from 1981 to 2010, centered on 1995, and provide information on average minimum and maximum temperatures, precipitation, and monthly relative humidity, representing historical climatic conditions [40].

Projections for future scenarios (2050, 2080, and 2100) were based on the A2 SRES scenario and the CSIRO-Mk3.0 (CS) Global Climate Model (GCM) from the Australian Climate Research Centre [41]. The CS model predicts a temperature increase of 2.11°C and a 14% reduction in precipitation [42]. The A2 SRES scenario accounts for moderate increases in greenhouse gas emissions due to population growth, economic development, and technological changes [43]. Using the A2 scenario to predict biological invasions enables the assessment of ecosystem vulnerability under extreme climatic conditions, aiding in developing robust and effective mitigation and adaptation strategies. The model’s reliability was assessed by comparing the projected potential distribution, as indicated by the Ecoclimatic Index (EI), with occurrences of *I. cylindrica* in Asia, its native region.

## Results

A total of 6.414 occurrences of *I. cylindrica* were documented, spanning North America, South America, Europe, Africa, Asia, the Middle East, and Australia (Fig 1a). According to the EI, approximately 16.4% of the global area is highly suitable for the species (EI ≥ 30), 20.0% is moderately suitable (0 <EI < 30), and 63.6% is unsuitable (EI = 0). These results include regions where *I. cylindrica* is considered native.

The globally modeled climatic suitability for *I. cylindrica* encompasses areas in Central America, South Africa, Australia, Asia, and southern Europe (Fig 1ab). Regions such as Central America, the southern United States, Asia, South Africa, the Middle East, New Zealand, Madagascar, and Pacific volcanic islands such as Samoa and Fiji were considered highly favorable for the species. In contrast, cold temperate zones, including Northern Europe, Canada, Russia, and the northern United States, were deemed unsuitable for *I. cylindrica* (Fig 1b).

**Fig 2:**
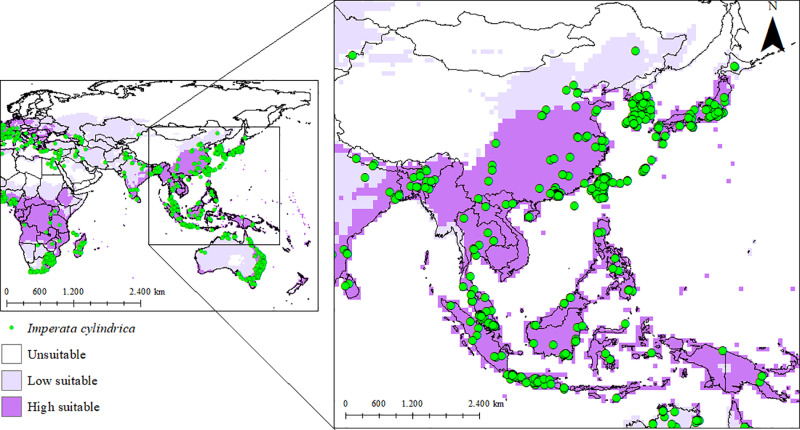
Current distribution of *Imperata cylindrica* in a validation region based on the Ecoclimatic Index (EI). Unsuitable areas in white (EI = 0), low-suitability areas in purple (0 <EI <30), and high-suitability areas in purple (30 ≤ EI ≤ 100).

The Asian region was selected for model validation due to the native occurrence of *I. cylindrica* and the high prevalence of the species, indicating climatic suitability in the area. Approximately 91% of the occurrence points in this region are in highly suitable areas, while 6% are in moderately suitable areas, and 3% are in unsuitable areas. These results validate and demonstrate the reliability of the final model (Fig 2).

The sensitivity analysis revealed that, for changes in unsuitable areas, the most sensitive model parameter was DV0; and for low and highly suitable areas, the temperature-related parameters (DV0, DV1, DV2, and DV3) and moisture-related parameters (SM0, SM1, SM2, and SM3) were the most influential. It was also observed that cold stress (TTCS) causes changes in moderately and highly suitable regions (Supplementary material).

**Fig 3:**
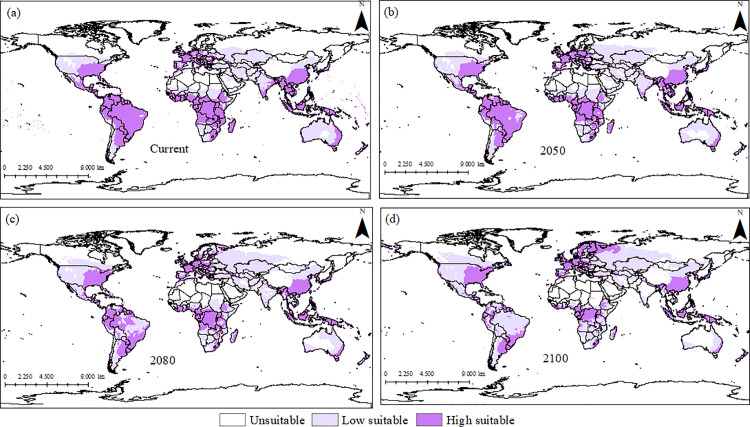
Current (a) and projected Ecoclimatic Index (EI) using Climex under CSIRO SRES A2 scenario for the years 2050 (b), 2080 (c) and 2100 (d), for *Imperata cylindrica.* Unsuitable areas in white (EI = 0), low-suitability areas in purple (0 <EI <30), and high-suitability areas in purple (30 ≤ EI ≤ 100).

The climate change model projects an increase of up to 19% in globally suitable areas for *I. cylindrica* by 2050, 2080, and 2100 (Supplementary material). In South America, climatically favorable areas for *I. cylindrica* increase substantially in southern Argentina, while they decrease in Ecuador (Fig 3). Under the projected scenario, warming significantly reduces climatically suitable areas for *I. cylindrica* in Africa, including Madagascar, Mozambique, southern Angola, northern Morocco, northern Libya, Oman, and Yemen. However, the rest of Africa remains suitable for the species (Fig 3).

**Fig 4:**
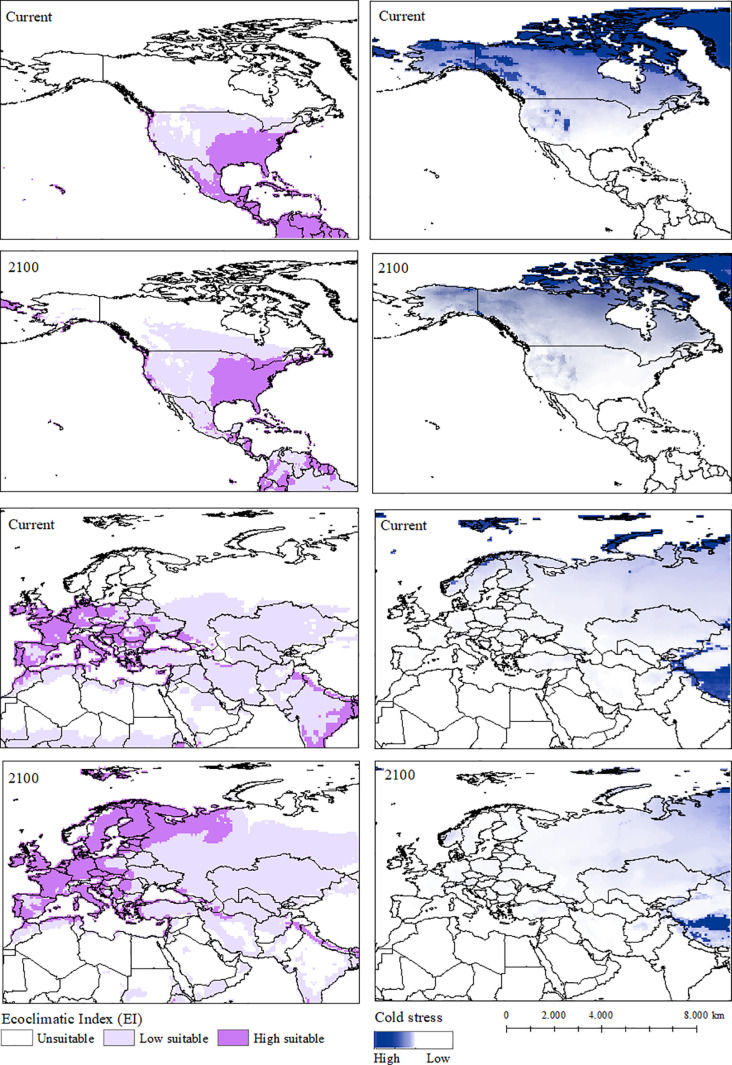
Current and projected Ecoclimatic Index for 2100 (EI) and, cold stress patterns using CLIMEX under the CSIRO SRES A2 scenario for *Imperata cylindrica* for North America and Europe. Unsuitable areas in white (EI = 0), low-suitability areas in purple (0 <EI <30), and high-suitability areas in purple (30 ≤ EI ≤ 100).

A moderate increase in climatically suitable areas for *I. cylindrica* is projected in North America. In Europe, there is a considerable expansion in the potential distribution range under climate change scenarios, particularly in countries such as Spain, Ukraine, Poland, and Germany (Fig 4).

## Discussion

Climate change may create conditions that favor introducing new invasive species into habitats where suitability has improved while altering the local distribution and abundance of existing native species [44]. Rising temperatures may also promote the expansion of species to higher latitudes, facilitating invasion or leading to significant loss of tropical species [45]. For *I. cylindrica*, potential distribution is primarily limited by temperature and drought, correlating with the Köppen climate classification. Highly suitable regions are concentrated in tropical (A), humid subtropical (Cfa), and Mediterranean (Csa and Csb) climates[46,47]. In contrast, unsuitable regions are associated with temperate climates (Dfa, Dfb, and Dfc), where long, cold winters result in temperatures often below the cold stress limit, limiting the occurrence of *I. cylindrica* [46,47].

With climate change, temperatures are expected to rise, leading to greater climatic suitability for *I. cylindrica* in countries such as Argentina and Canada, while regions like Ecuador may experience temperatures unsuitable for its growth [48–50]. Such distribution patterns are typical for tropical C4 species [26,51]. Prolonged low temperatures during winter increase photorespiration and reduce the efficiency of C4 metabolism; consequently, these species become less competitive in cold environments, explaining their absence in such regions. Nonetheless, the phenotypic plasticity of *I. cylindrica* allows the species to at least partially overcome these barriers [52–55].

Results regarding the potential distribution of species like *I. cylindrica* are crucial for identifying priority areas for monitoring, prevention, and management. Implementing an early warning system can facilitate swift interventions, minimizing spread. SDMs consider climate as the primary determining factor in the potential distribution of *I. cylindrica*; this limitation does not invalidate the results, but it is essential to acknowledge it [11]. SDMs disregard biotic interactions, such as competition, predation, mutualisms, and the presence of pathogens, which can significantly influence population dynamics. Furthermore, the exclusion of local abiotic factors, such as topography, nutrient availability, and edaphic conditions, may lead to the overestimation or underestimation of environmental suitability [31,56].

Regarding the correlationist model proposed by Bradley et al. (2010) [19], the predicted areas suitable for *I. cylindrica* correspond to locations we identified as highly suitable. However, there is an increase in current and predicted moderately suitable areas by 2100, as shown in Fig 4. Compared to the model proposed by Ray et al. (2019) [20] for northeastern India, our results show a decrease in current and future suitability. In this context, mechanistic models may overestimate or underestimate potential invasion areas [11].

Moreover, the selection of different climate scenarios can significantly impact the potential distribution of the species. The A2 scenario, widely used in species modelling studies, projects a more hostile future. In contrast, the A1B scenario assumes moderate emissions, presenting a less severe climatic context [57]. The model proposed for *I. cylindrica* using CLIMEX under the A2 SRES scenario encompasses larger areas susceptible to invasion and has a global scope, unlike models restricted to specific regions or countries. This broader scope is crucial for predicting invasion patterns, allowing for identifying risk areas that could be overlooked in regional analyses. Moreover, overestimating potential invasion areas may prove advantageous, as it enables the implementation of more comprehensive and proactive prevention and mitigation strategies, typically associated with lower financial investments. Thus, our findings contribute to a global projection of *I. cylindrica* distribution, providing valuable insights into ecosystem vulnerability and highlighting the most influential climatic parameters in determining the species’ distribution.

## Conclusion

Climate change, biological invasions, and anthropogenic disturbances are significant drivers of global environmental change, and their complex interactions shape the functioning of ecosystems, societies, and economies. This study underscores the importance of understanding distribution patterns and the impacts of climate change on the spread of invasive species such as *Imperata cylindrica*. The invasive potential of *I. cylindrica* is significantly influenced by climatic factors, particularly temperature and precipitation regimes. With the projected increase in global temperatures, this species is expected to expand its range into new regions, posing further challenges for ecosystem and natural resource management.

Ongoing research and the development of adaptive management strategies are essential to address the emerging challenges associated with the spread of invasive species in the context of climate change. Accurately delineating a species’ geographical distribution is difficult and relies on the complex interaction of various factors. While SDMs, such as CLIMEX, are valuable tools for predicting the potential spread of invasive species, it is crucial to acknowledge their limitations. These models are based on climatic variables and do not account for biotic and abiotic interactions, which also play a key role in species distribution. Therefore, integrating long-term field studies, remote sensing data, and ecological interaction analyses is recommended to enhance prediction accuracy and reduce uncertainties.

## Supporting information

S1 Fig Changes in unsuitable areas (EI = 0) (a), low-suitability areas (0 <EI < 30) (b), and high-suitability areas (30 <EI < 100) (c), in %, for *Imperata cylindrica*., in a sensitivity analysis using CLIMEX, based on parameters of greater sensitivity for the Ecoclimatic Index (EI). The values for the parameters used are shown in Table 1.
(TIFF)
