# Supplementary figures and images for: Predicting the spread of invasive Imperata cylindrica under climate change: A global risk assessment and future distribution scenarios
Source: PLoS One. 2025 May 9;20(5):e0321027. doi: 10.1371/journal.pone.0321027 (PMC12063829; doi:10.1371/journal.pone.0321027)

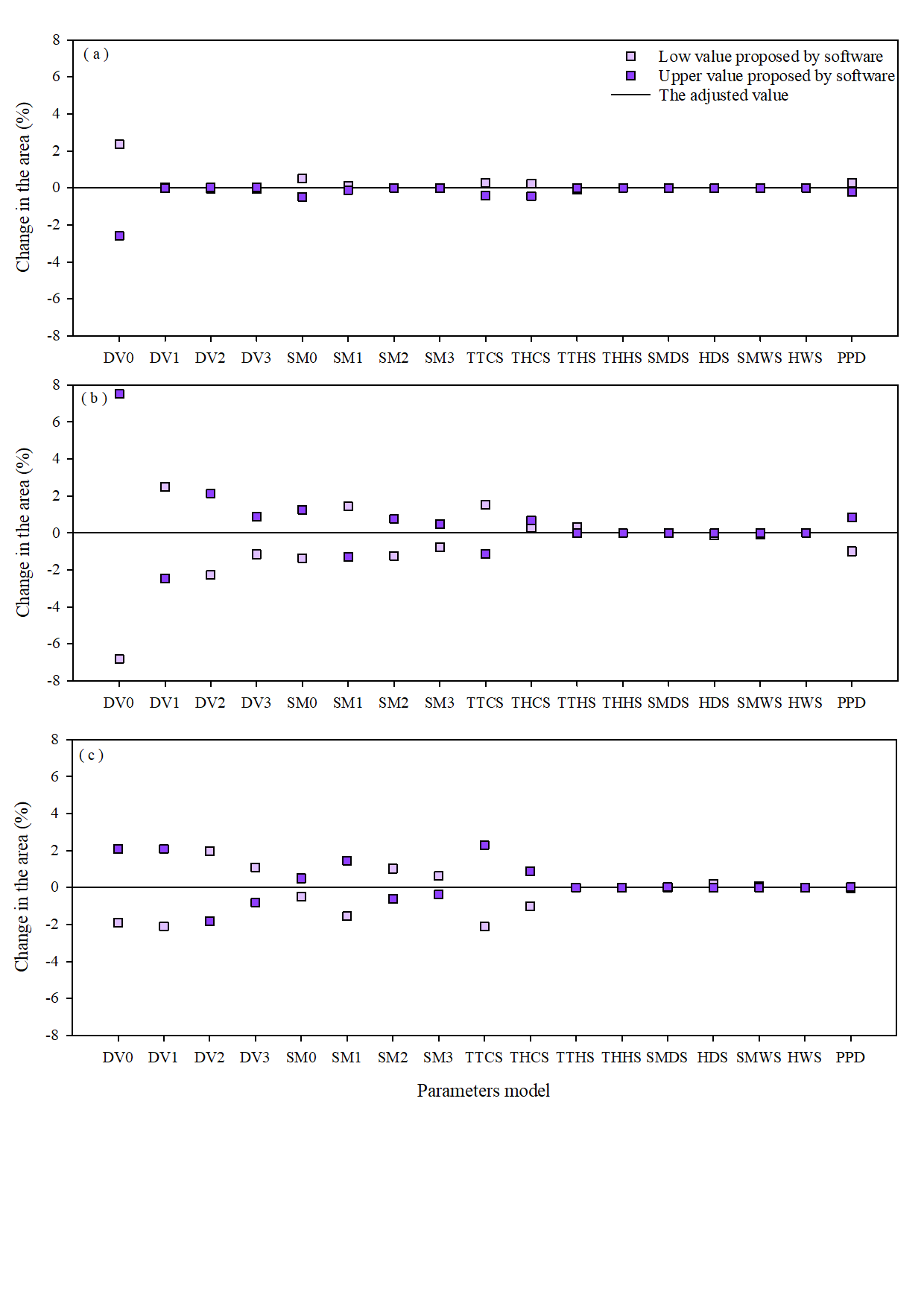

Supplement: S1 Fig — The values for the parameters used are shown in Table 1. (TIFF) [file pone.0321027.s001.tif]
